# Regeneration of Osteochondral Lesion of the Talus with Retrograde Drilling Technique: An In Vitro Pilot Study

**DOI:** 10.3390/jcm13144138

**Published:** 2024-07-16

**Authors:** Francesca Veronesi, Melania Maglio, Silvia Brogini, Antonio Mazzotti, Elena Artioli, Simone Ottavio Zielli, Cesare Faldini, Gianluca Giavaresi

**Affiliations:** 1 Surgical Sciences and Technologies, IRCCS Istituto Ortopedico Rizzoli, Via di Barbiano 1/10, 40136 Bologna, Italy; francesca.veronesi@ior.it (F.V.); silvia.brogini@ior.it (S.B.); gianluca.giavaresi@ior.it (G.G.); 2 1st Orthopaedics and Traumatologic Clinic, IRCCS Istituto Ortopedico Rizzoli, Via Putti 1, 40136 Bologna, Italy; antonio.mazzotti@ior.it (A.M.); elena.artioli@ior.it (E.A.); simoneottavio.zielli@ior.it (S.O.Z.); cesare.faldini@ior.it (C.F.); 3 Department of Biomedical and Neuromotor Sciences (DIBINEM), Alma Mater Studiorum University of Bologna, 40126 Bologna, Italy

**Keywords:** retrograde drilling, osteochondral lesions, talus, in vitro model, tissue culture model, histology, microCT imaging

## Abstract

**Background:** Retrograde Drilling (RD) is a surgical technique employed for osteochondral lesions of the talus (OCLTs) to reach the subchondral bone lesion from behind, thus preserving cartilage integrity. The aim of the present pilot study was to set up an in vitro model of OCLTs to evaluate the regenerative potential of biological approaches that could be associated with the RD technique. **Methods:** For this purpose, an OCLT was created in human osteochondral specimens, to try to mimic the RD technique, and to compare the regenerative potential of two biological treatments. For this purpose, three groups of treatments were performed in vitro: (1) no treatment (empty defect); (2) autologous bone graft (ABG); (3) hyaluronic membrane enriched with autologous bone marrow cells. Tissue viability; production of Collagen I and II, Vascular Endothelial Growth Factor, and Aggrecan; and histological and microCT evaluations were performed after 30 days of culture in normal culture conditions. **Results:** It was observed that Group 3 showed the highest viability, and Group 2 showed the highest protein production. From a histological and microtomographic point of view, it was possible to appreciate the structure of the morcellized bone with which the defect of Group 2 was filled, while it was not yet possible to observe the deposition of mineralized tissue in Group 3. **Conclusions:** To conclude, this pilot study shows the feasibility of an alternative in vitro model to evaluate and compare the regenerative potential of two biological scaffolds, trying to mimic the RD technique as much as possible. The tissues remained vital for up to 4 weeks and both ABG and hyaluronic acid-based scaffolds stimulated the release of proteins linked to regenerative processes in comparison to the empty defect group.

## 1. Introduction

Osteochondral lesions of the talus (OCLTs) involve subchondral bone (SB) and the overlying cartilage, principally affecting active and young patients. OCLTs mainly result from traumatic injuries, such as ankle sprains or fractures, which can disrupt the smooth surface of the cartilage and cause pain and swelling. If left untreated, these lesions can lead to long-term joint damage and degenerative osteoarthritis (OA) [1]. The necessity for surgery depends on the severity and symptoms of the lesion; however, due to the poor regenerative properties of cartilage, surgical treatment is often required [2,3]. Among treatment options, drilling is well established due to its simplicity and cost-effectiveness, to promote bone formation by stimulating angiogenesis. Drilling can be performed in anterograde or retrograde fashion, based on the direction of the drill toward the lesion [4].

Anterograde Drilling (AD) was described for the first time by Kumai et al. in 1999 [5]. This technique is characterized by the insertion of a K-wire directly into the lesion through the cartilage. This approach has several weaknesses: the wire passes through the intact cartilage, causing epiphyseal line injury [5,6] and leading to heat generation. Moreover, AD does not allow us to reach the dorsomedial aspect of the talar dome.

On the contrary, Retrograde Drilling (RD) allows us to reach SB lesions from behind, thus preserving cartilage integrity. Described for the first time by Lee and Mercurio in 1981 [7], RD is mainly employed in the presence of SB lesions with intact overlying articular cartilage [3]. RD is also applied when it is difficult to reach the lesion using other anterograde approaches, either arthrotomy or arthroscopic [4,8]: the defect should be targeted accurately, because the SB should be revascularized and new bone should be formed [9]. Its principal advantages are the possibility of drilling close to the SB using intraoperative fluoroscopy, without damaging articular cartilage, and its minimally invasive nature, which does not require an osteotomy [10,11,12].

A recent systematic review on the use of RD treatment for OCLTs over the past ten years highlighted the presence of very few clinical data, despite the promising outcomes, as concerns remain about the regenerative potential of the SB, especially in the presence of large lesions or cysts [13,14]. The use of autologous bone graft (ABG), as a biological adjuvant to increase the effectiveness of RD in terms of regenerative burst, has given mixed results over the past 10 years, even with the combined use of further elements, like collagenous membrane [15,16,17,18]. Far from having found the optimal approach to improve the RD technique, the use of advanced in vitro models with human tissue might be currently considered one of the best options to study the biological response of tissues to new treatment combinations.

In this regard, the present pilot study aimed to set up an in vitro model of OCLTs to evaluate the regenerative potential of biological approaches that could be associated with the RD technique. For this purpose, osteochondral (OC) samples from patients undergoing ankle prosthesis were used to compare the regenerative potential of two biological adjuvants, ABG and a hyaluronic membrane enriched with autologous bone marrow cells, trying to mimic different RD techniques.

## 2. Materials and Methods

### 2.1. OC Sample Harvesting and Surgical Set-Up of OCLT

This study was approved by the Local Ethics Committee of the Emilia Romagna Region (Comitato Etico Indipendente Area Vasta Emilia Centro, CE-AVEC, number: 802/2022/Sper/IOR) and was carried out in accordance with relevant guidelines and regulations (IRCCS Istituto Ortopedico Rizzoli has kept the ISO 9001 [19] Quality certification since 2008, with special reference to the research area). OC specimens were harvested from patients undergoing surgery for Total Ankle Arthroplasty following collection of written informed consent.

Inclusion and exclusion criteria are listed in Table 1.

OC samples were obtained from the distal tibia as waste material from 15 patients, 9 males and 6 females (mean age of 61.8 ± 8.7 years). A defect of 5 mm width and 4 mm height was created, through the RD technique, to mimic an OCLT in all specimens. The size of the defect was measured considering the thickness of the talar resection (4 mm) according to the surgical technique and using the 5 mm wide perforator available in the dedicated instrumentation. These dimensions were considered to relate to typical clinical scenarios. The specimens were then divided according to the treatment as follows:

- Group 1—control untreated (CTR): the defect created was left empty and not treated (empty defect);
- Group 2—treated with ABG: to simulate the clinical surgical procedure, for bone drainage with ABG, the technique developed by Taranow WS et al. was used [20], a method widely validated in the literature which proved to be indicated in the presence of subchondral cystic lesions with vital cartilage [4,14,16]. The created defect was filled with the obtained spongy bone graft, deposited beneath the subchondral plate and adequately packed to prevent it from leaving the defect.
- Group 3—involved a similar retrograde perforation, removing all the subchondral bone and keeping the overlying cartilaginous layer intact, but the defect was treated with the HyaloFast^®^ (Anika Therapeutics, Inc.—Bedford, MA 01730 USA) scaffold: HYAFF^®^ (hyaluronic acid benzyl ester) is a scaffold usually used as a support for the entrapment of mesenchymal stem cells (MSCs) and for the repair of chondral and osteochondral lesions. The created defect was filled with HyaloFast^®^ scaffolds and bone marrow cells harvested from the tibial medullary canal emerging through the tibial holes of the surgical instruments (2 mL) during the preparatory phase for the positioning of the prosthesis.

Human samples were harvested during prosthetic surgery and all the procedures to shape the specimens, to create the defects and to place materials related to the treatment—when required—were performed in the operating room, thus ensuring the sterility foreseen for clinical surgical procedures. All the samples were placed in sterile boxes in physiologic solution, to keep tissue hydrated, and transported to the laboratory immediately after the end of the surgery, to put samples in the culture medium suitable for preserving the viability. Once in the laboratory, all the procedures—set-up of culture, viability tests, medium change—were performed under a biological laminar flow hood, guaranteeing the preservation of sterility.

### 2.2. Culture Conditions

OC samples were then placed individually in a sterile TubeSpin Bioreactors, where gas exchange was ensured by a screw cap with a 0.22 μm filter membrane (TPP TubeSpin^®^ Bioreactors, TPP Techno Plastic Products, Trasadingen, Switzerland). TubeSpin Bioreactors were immediately placed on a rolling apparatus with a 20° inclined plane, at 5 rpm (Thermo Scientific, Waltham, MA, USA), and cultured for up to 4 weeks at 37 °C in a 5% CO_2_/95% air-controlled atmosphere.

Each tube was filled with culture medium that consisted of Dulbecco’s Modified Eagle’s Medium (DMEM, Sigma-Aldrich, St. Louis, MO, USA) supplemented with 5 mg/mL of bovine serum albumin (BSA, SigmaAldrich, USA), 2 mM glutamine, and antibiotics (100 U/mL penicillin, 100 μg/mL streptomycin) (Gibco, INVITROGEN Corporation, Waltham, MA, USA) and with no fetal calf serum. Culture medium was changed every 3 days.

### 2.3. Tissue Viability

Tissue viability was evaluated at the time of tissue culture (T0), and after 2 (T2) and 4 (T4) weeks of culture. Three wells for each patient were used to assess tissue viability.

The Alamar Blue test was performed. More precisely, Alamar Blue dye (Serotec, Oxford, UK) was added to the media of samples and to the media alone as control (1:10 *v*/*v*) and incubated for 4 h at 37 °C in the dark, in accordance with the manufacturer’s instructions. After transferring the supernatants to 96-well plates, the absorbance of supernatants was read spectrophotometrically using a fluorescence excitation wavelength of 570 nm (excitation range of 540–570 nm) and an emission wavelength of 600 nm (emission range of 580–610 nm) (for the fully oxidized and reduced forms of the reagent) by a microplate reader (BioRad, Hercules, CA, USA).

The results, obtained as optical density (OD) data, were processed following the manufacturer’s instructions. Viability measures were expressed as relative fluorescence units (RFU).

### 2.4. Immunoenzymatic Analysis

At T4, supernatants of all groups were collected and centrifuged to eliminate the particulates. Three wells for each patient were used for tests. Immunoenzymatic assays, enzyme-linked immunosorbent assays (ELISAs), were employed to quantify the amount of Collagen I (COLL I, ng/mL), Collagen II (COLL II, pg/mL), Vascular Endothelial Growth Factor (VEGF, pg/mL), and Aggrecan (ng/mL) (Cloud-Clone Corp., Houston, TX, USA) (pg/mL), according to the manufacturer’s instructions.

### 2.5. Microtomographic Analysis

At T4, immediately after culturing and before sample preparation for histological evaluation, samples were fixed in 10% buffered formalin for 24 h. Then, they were extensively rinsed in distilled water and immersed in 70% alcohol solution.

Samples were scanned using a high-resolution microCT system Skyscan 1172 (Bruker, Kontich, Belgium) at a source voltage of 70 kV and a source current of 140 μA, with the following scan settings: aluminum 0.5 mm filter, nominal resolution at 36 μm, rotation step of 0.7°, and a frame averaging of 4. The obtained dataset was reconstructed with NRecon software (version 1.7.4.6, Bruker, Belgium) applying corrections for specific misalignment and ring artifact reduction. Reconstructed images were oriented using Dataviewer software (version 1.7.0.1, Bruker, Belgium) to visualize the defect along its major axis.

**Figure 1:**
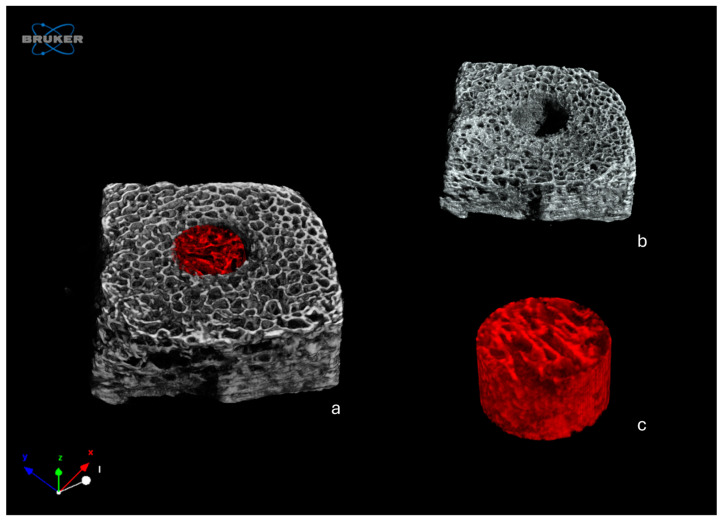
Identification of the VOI for the morphometric microCT analysis: (**a**) complete 3D rendering of a representative sample from Group 2, with the volume corresponding to the defect highlighted in red; (**b**) subtraction of the measured VOI from the 3D rendering of the sample; (**c**) 3D rendering of the VOI corresponding to the defect.

A volume of interest (VOI) corresponding to the created defect (about 2.5 mm height, 4 mm diameter) was built using CTAn software (version 1.20.3.0; Bruker, Belgium) and quantitative 3D analyses were carried out on the following morphological parameters (Figure 1):

- Bone density BV/TV (%), expressed as the ratio between the volume of the trabecular bone and the total volume of the VOI;
- Trabecular thickness Tb.Th (in mm), calculated in a model-independent manner described by Hildebrand and Ruegsegger on the entire VOI;
- Trabecular separation Tb.Sp (in mm), calculated as the Tb.Th.

Three-dimensional renderings of the analyzed samples were obtained with CTVox software (version 3.3.1, Bruker, Belgium) (Figure 1).

### 2.6. Histology

At T4, after microtomographic analysis, samples were processed for paraffin embedding. Briefly, samples were decalcified in a 5% solution of formic and hydrochloric acid for about 1 week. Then, they were extensively rinsed in distilled water, dehydrated in graded alcohol solutions (70%, 95%, two times, and 100%, three times, 60 min for each step), cleared in xylene, and finally embedded in paraffin. Five-micrometer-thick sections for each sample were obtained by a semi-automated microtome (Microm International GmbH, Heidelberg, Germany).

Three slides from each sample were dewaxed in decreasing graded ethanol solutions and then stained with Safranin O-Fast Green.

Images from stained slides were captured with a high-resolution digital pathology slide scanner (AperioScanscope AT2 System, Aperio Technologies, Vista, CA, USA) and evaluated at different magnifications to observe tissue morphology and structure after culture, cellularity, and possible signs of matrix deposition.

### 2.7. Statistical Analysis

Since this is an in vitro pilot study, power analysis was not performed and n = 5 samples per group (total n = 15) was defined.

Statistical analysis was performed with GraphPad Software, version 9.0.0 (San Diego, CA, USA). The results of assays are reported as mean ± standard deviations (SDs) at a significance level of *p* < 0.05. After having verified normal distribution and homogeneity of variance, a one-way or two-way (viability test) ANOVA was conducted to compare data among groups. Finally, the post hoc Tukey’s test was performed to detect significant differences among groups for the ELISA test and microtomographic analysis, while the Holm–Sidak test was used for viability test.

## 3. Results

### 3.1. Viability

**Figure 2:**
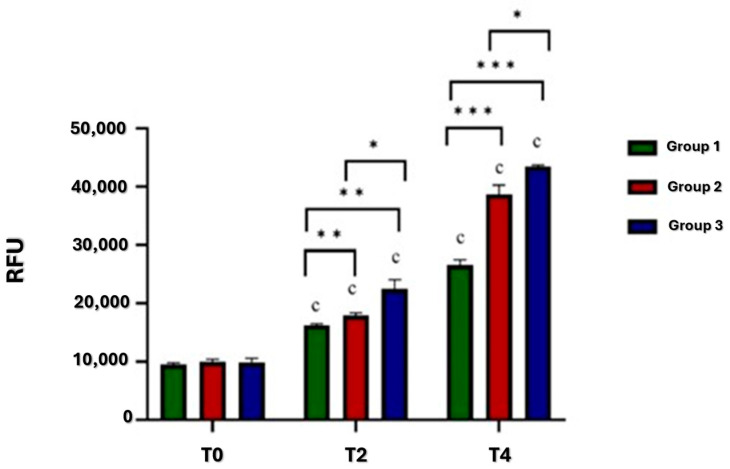
Histograms of osteochondral defect culture viability of Groups 1, 2, and 3. T2: ** Group 2 vs. Group 1 (*p* = 0.004); ** Group 3 vs. Group 1 (*p* = 0.006); * Group 3 vs. Group 2 (*p* = 0.01). T4: *** Group 2 and Group 3 vs. Group 1 (*p* < 0.001); * Group 3 vs. Group 2 (*p* = 0.01). c T2 vs. T0 and T4 vs. T2 (*p* < 0.001).

As shown in Figure 2, the viability of the tissues showed significant differences among the three groups and the three experimental times (T0, T2, T4).

As regards comparison among groups, at T0, no significant differences were observed.

At T2, Group 2 showed significantly higher viability in comparison to Group 1 (*p* = 0.004), and Group 3 in comparison to Group 2 (*p* = 0.01) and Group 1 (*p* = 0.006).

Similarly, at T4, Group 2 showed significantly higher viability than Group 1 (*p* < 0.001), and Group 3 than Group 1 (*p* < 0.001) and Group 2 (*p* = 0.01).

As regards comparison among experimental times, the viability of all groups increased, from T0 to T2 (*p* < 0.001) and from T2 to T4 (*p* < 0.001).

### 3.2. Immunoenzymatic Analysis

**Figure 3:**
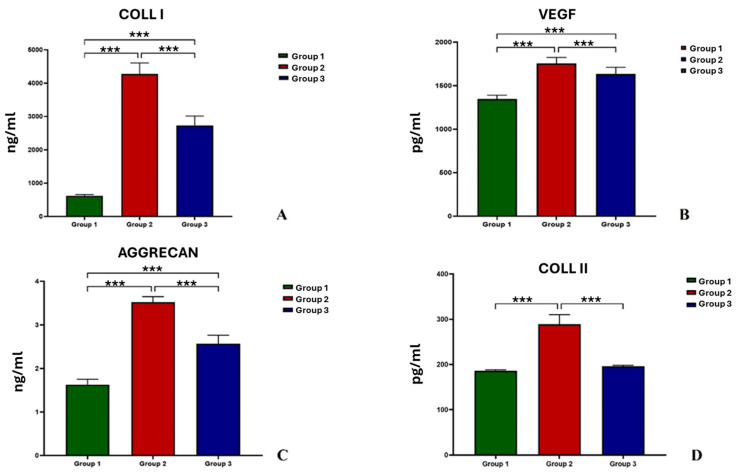
Histograms of COLL I (**A**), VEGF (**B**), Aggrecan (**C**), and COLL II (**D**) of Groups 1, 2, and 3. COLL I, VEGF, Aggrecan: ***, Group 2 and Group 3 vs. Group 1 (*p* < 0.001); Group 2 vs. Group 1 (*p* < 0.001). COLL II: ***, Group 2 vs. Group 1 and Group 3 (*p* < 0.001).

As observed in Figure 3, COLL I (Figure 3A), COLL II (Figure 3B), VEGF (Figure 3C), and Aggrecan (Figure 3D) showed significant differences among groups at T4.

COLL I, VEGF, and Aggrecan showed higher values in Group 2 than in Group 1 and Group 3 (*p* < 0.001), and in Group 3 than in Group 1 (*p* < 0.001) (Figure 3A–C).

COLL II was significantly higher in Group 2 in comparison to Group 1 and Group 3 (*p* < 0.001). No significant differences were observed between Group 1 and Group 3 (Figure 3D).

### 3.3. Microtomographic Analysis

**Figure 4:**
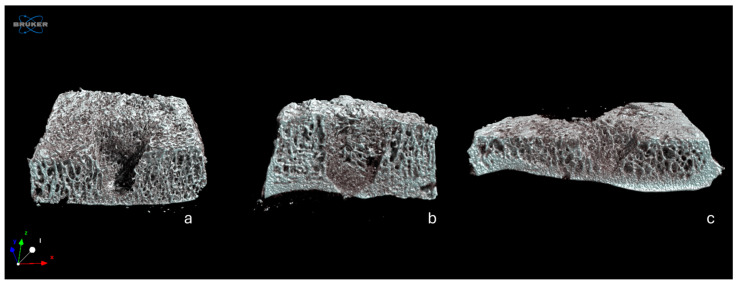
Representative 3D rendering of samples from Group 1 (**a**), Group 2 (**b**), and Group 3 (**c**).

The microtomographic images confirmed that the surgically created defect did not exceed the boundary of the cartilaginous articular surface, and that in the group in which the defect was filled with ABG, the material remained in place (Figure 4).

**Figure 5:**
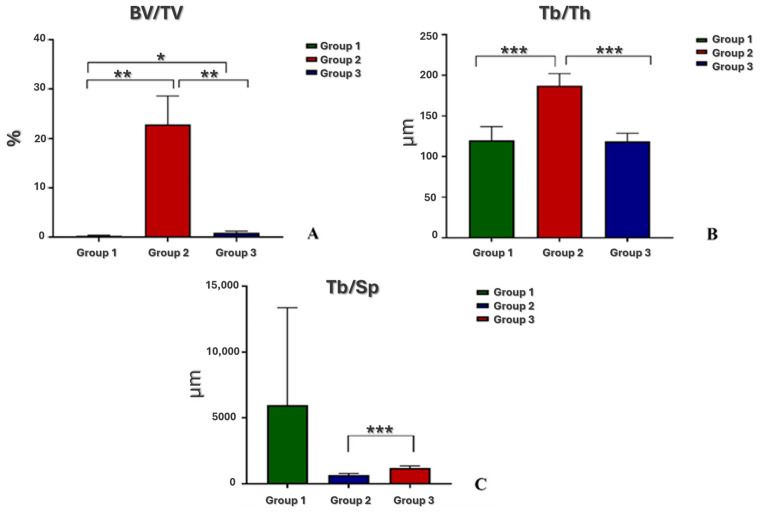
Histograms of microtomographic parameters BV/TV (**A**), Tb.Th (**B**), and Tb.Sp (**C**) of Groups 1, 2, and 3 (n = 3 replicates). BV/TV: *, Group 3 vs. Group 1 (*p* = 0.03); **, Group 2 vs. Group 1 (*p* = 0.002); **, Group 2 vs. Group 3 (*p* = 0.003). Tb.Th: ***, Group 2 vs. Group 1 and Group 3 (*p* < 0.001). Tb.Sp: ***, Group 3 vs. Group 2 (*p* < 0.001).

At T4, BV/TV, Tb.Th, and Tb.Sp showed significant differences among groups (Figure 5A–C).

As regards the BV/TV parameter, Group 2 showed higher values than Group 1 (*p* = 0.002) and Group 3 (*p* = 0.003), and Group 3 than Group 1 (*p* = 0.03).

Tb.Th was significantly higher in Group 2 in comparison to Group 1 and Group 3 (*p* < 0.001), while no significant differences were observed between Group 1 and Group 3.

Finally, Tb.Sp was significantly lower in Group 2 in comparison to Group 3 (*p* < 0.001), and no significant differences were found between Group 1 and the other groups.

### 3.4. Histology

**Figure 6:**
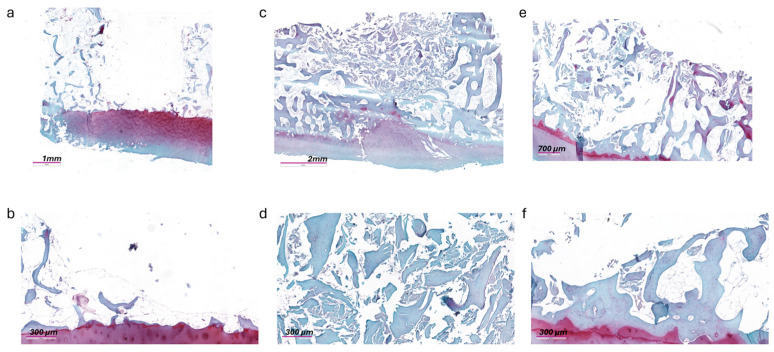
Histological overview of Group 1 (**a**), Group 2 (**c**), and Group 3 (**e**). Magnification 2×. Safranin O/Fast Green staining. Details of interface between articular cartilage, surrounding bone, and defect of Group 1 (**b**) and Group 3 (**f**) and of defect filled with ABG in Group 2 (**d**). Magnification 20×. Safranin O/Fast Green staining.

Representative histological images of the samples from each experimental group at the end of in vitro culture are shown in Figure 5. In the samples from Group 1, with the bone defect left empty, no signs of matrix deposition are observed in the areas at the interface between the residual bone and the defect area, suggesting that a process of spontaneous regeneration was not established (Figure 6a,b). The ABG used to fill the defect in Group 2 remained in place, supporting the structure and conformation of the surrounding tissue without interposition of fibrous connective tissue (Figure 6c,d).

In samples from Group 3, the presence of bone spicules can be observed inside the area of the defect in the absence of a structured organization of the tissue or integration with the pre-existing surrounding bone (Figure 6e,f).

At higher magnification (20×), in Group 2 and Group 3, the presence of full osteocyte lacunae and preservation of the lamellar structure can be observed, in continuity with the surrounding bone, as well as dense and irregular connective tissue.

As regards overlying cartilage tissue, the histology confirmed the presence of intact tissue, with signs of osteoarthritic features, in accordance with the clinical picture of donor patients, going from fibrillations and clefts to a fibrocartilaginous formation.

## 4. Discussion

The use of scaffolds for the treatment of OCLTs is consolidated in surgical practice; in fact, scaffolds provide essential support and a framework for new tissue growth, enhancing mechanical stability and stimulating chondrogenic differentiation, thereby promoting tissue repair and regeneration. The choice of the scaffold (mainly biosynthetic, biological, and hydrogel) depends on the specific characteristics of the lesion, the patient’s condition, and the surgeon’s preference. Among the available options, biological scaffolds, derived from natural sources like Collagen or extracellular matrix components, provide a biological framework that promotes cell attachment, migration, and proliferation [21,22].

In the literature from the last 10 years, ABG has been the most employed technique [17], often associated with a membrane based on COLL I/COLL III [18], while hyaluronic-based scaffolds are still rarely used [23]. In several clinical studies, these types of scaffolds have been shown to improve joint function and reduce pain in patients affected by OCLTs, but there are few studies comparing the outcomes of different approaches [17,18,23].

In this context, the use of an advanced in vitro model turns out to be particularly useful to test different treatment options and carry out preliminary evaluations on the most promising combinations.

The in vitro model, developed in the present study, was set up to culture human osteochondral tissues in which OCLTs were recreated and evaluate the in vitro responses to scaffold-based treatments, a hyaluronic acid membrane with autologous bone marrow cells and ABG, trying to mimic different RD techniques used in clinical practice. The evaluation of the outcome, in comparison to the untreated (empty) defect, was conducted with different methods to evaluate the preservation of tissue viability, the release of proteins related to the onset of regenerative processes, and the morphology and structure of the tissue.

Data collected from the culture confirmed that the ABG lends itself to acting as a scaffold for the growth of bone tissue. ABG, which is used mainly for the reintegration of the structure and the bone conformation, contributing to the preservation of the mechanical characteristics and stimulating new bone formation, remained at the site of implantation in the tissue cultured in vitro, maintaining a native structure and morphology and favoring the release of factors such as COLL I and II, Aggrecan, and VEGF.

These factors play a critical role in the structure, function, and repair processes of bone and cartilage. COLL I and II and Aggrecan are involved in effective joint repair and long-term functionality, providing structural support and strength to the SB for load bearing and shock absorption, supporting chondrocyte function [24,25]. By ensuring a well-vascularized and supportive environment, VEGF significantly contributes to the successful regeneration of the osteochondral unit, recruiting stem cells to the site of injury and promoting their differentiation into osteoblasts and chondrocytes, also modulating the inflammatory response [26].

The higher values of these key factors when using the hyaluronic acid-based scaffold in comparison to the control (empty) group, in particular of COLL I, VEGF, and Aggrecan, suggest that the presence of the HyaloFast^®^ membrane, implemented with bone marrow cells, exerts a stimulus on the surrounding bone tissue that promotes reparative and regenerative processes.

Hyalofast^®^ is a biodegradable hyaluronic acid-based membrane, already employed in clinical practice, whose fibers favor the adhesion and arrangement of cells to promote osteochondral regeneration. Its design makes it adaptable to being inserted into defects of various sizes, making it particularly suitable for use in the present in vitro model [27].

The positive and trophic effects of the in vitro-tested approaches for the treatment of OCLTs compared to the untreated group are reflected not only in the immunoenzymatic results but also in the viability trend. Over the four weeks of culture, viability increased in all groups, with greater peaks in the ABG and Hyalofast^®^ samples. The comparable viability values found in the test at time zero suggest homogeneity between the samples and that the differences observed over the experimental time may be linked to the treatments. In vitro literature showed that Hyalofast^®^ was used for the commitment of bone marrow mesenchymal stem cells (BMSCs), after 35 days of culture, acquiring an osteogenic or chondrogenic phenotype depending on the presence of specific differentiation-inducing factors added into the culture medium [28]. Instead, in the present in vitro study, the production of the OC unit factors, after 30 days of culture, was observed even without differentiation stimuli present in the culture medium.

The histological aspect of the cultured tissues, after 4 weeks of culture, confirmed the patient inclusion criteria of the protocol, which were being affected by primary or secondary osteoarthritis of the ankle (KL 4), but having an intact cartilaginous layer, all features that predispose a patient to be eligible for the RD surgical technique. Histological results support these findings, albeit within the limits of the reorganization processes observable in a medium-term cultivation model. This is also evident in the microCT results, where, in the group with ABG, it is possible to appreciate the structure of the morcellized bone with which the defect is filled, while it is not yet possible to observe the deposition of mineralized tissue in the group treated with Hyalofast^®^. However, the evaluation of the data as a whole suggests a promising start of stimulation in a regenerative sense, which, in more complex systems and with longer experimental times, could lead to observing the formation of new tissue.

In preclinical orthopedic research, animal models are still commonly used for the evaluation and comparison of new scaffolds and surgical techniques for regenerative approaches; however, beyond the ethical concerns and increasingly higher costs, the different regenerative capacities of some in vivo models, sometimes very distant from human ones, can make it difficult to make a correct prediction of clinical outcomes of the tested materials [29]. Thus, the development of advanced in vitro models, able to recreate living microenvironments exploiting the use of human tissues, has become necessary in recent years.

In the field of orthopedic regeneration, most in vitro models regard cancer metastases [30], while few studies have employed the culture of osteochondral defects for the first step of regenerative medicine [31].

Our group has previously set up an in vitro model for the evaluation of osseointegration and bone ingrowth of titanium alloy [32,33]. Rabbit cortical bone segments were cultured with titanium alloy cylinders, showing for the first time the validity of this alternative in vitro method [32]. Subsequently, a preliminary comparison between two combinations of COLL coverings for titanium alloy implants, in terms of cell adhesion, viability, and bone matrix production, for probable future use as a bone implant, was performed in vitro. An initial bone matrix deposition was observed, especially in the presence of the two coatings [33].

In our opinion, OCLT tissue culture, which refers to the cultivation of entire tissue segments in a controlled environment, is a crucial step before in vivo evaluation for several reasons. First, in toto tissue culture allows researchers to observe cellular behaviors, such as proliferation, differentiation, and matrix production, without the complexities and variables present in an in vivo system. It allows for the early detection of potential issues, such as cytotoxicity, inadequate cell integration, or poor mechanical stability, and helps optimize the interaction between scaffolds and cells, crucial for the success of the tissue construct once it is implanted. In addition, by thoroughly testing tissue constructs in vitro, the need for extensive animal testing can be reduced. This aligns with ethical guidelines aimed at minimizing the use of animals in research. Indeed, in vitro tissue cultures allow for the refinement of the constructs before moving to in vivo models, potentially reducing the number of animals required for meaningful results. In vitro cultures provide preliminary data that can support preclinical validation.

The present study has some limitations: (1) The in vitro evaluations were performed after 4 weeks of culture, which allowed an overall evaluation of culture, viability, and protein release but still not the regeneration of the architecture of the defect; with longer experimental times, it might be possible to observe a better regenerative potential of the hyaluronic membrane enriched with bone marrow cells. (2) Although, in reality, this membrane has always been used as a covering for cartilaginous and osteochondral defects, this study has shown promising results even if it is used as a filler for bone defects. (3) No histological and microCT evaluations of OCLTs were performed at T0. In future, further analysis will be performed, such as immunohistochemistry and histomorphometry, and the experimental times will be lengthened.

Clinical implications and future research directions should focus on the hyaluronan scaffold rich in nucleated cells positioned under the pathological cartilage and on its behavior over time. In the case of subchondral cysts, a poor healing response may occur because of the non-viable subchondral bone and scarcity of MSCs for restoring the lesions. The hyaluronan scaffold soaked with the bone marrow cells harvested from the medullary canal may represent an important source of MSCs, therefore promoting cell interaction and mechanical stability during the regenerative process.

## 5. Conclusions

In this pilot study, the feasibility of an alternative in vitro model for evaluating and comparing the regenerative potential of two biological scaffolds, ABG and a hyaluronic acid-based scaffold enriched with autologous bone marrow cells, was assessed, trying to mimic the RD surgical technique as much as possible, used in the surgery room for some OCLTs.

It was confirmed that the tissues remained vital for up to 4 weeks, opening the prospect for the set-up of long-term cultures, and that both ABG and hyaluronic acid-based scaffolds stimulated the release of proteins linked to regenerative processes in comparison to the empty defect group. However, longer experimental times are needed to be able to observe progressions in the regenerative process in a complex microenvironment such as OCLTs in vitro.

## Figures and Tables

**Table 1:** Inclusion and exclusion criteria of patients from which osteochondral tissues were harvested.

Inclusion Criteria	Exclusion Criteria
Male and female patients suffering from primary or secondary arthritis of the ankle (Kellgren–Lawrence—KL 4)	Presence of active infections at the time of inclusion in the study
Thickness of waste osteocartilaginous tissue ≥ 3 mm	State of pregnancy
Age ≥ 18 years	Any other pathology or state that makes surgical treatment contraindicated
Patients able to provide written informed consent to the study	Patients suffering from autoimmune or rheumatological diseases

## Data Availability

The original contributions presented in the study are included in the article; further inquiries can be directed to the corresponding author/s.
